# Supplementary material for: Implementing Standardized Patient Caregivers to Practice Difficult Conversations in a Pediatric Dentistry Course
Source: MedEdPORTAL. 2022 Jan 3;18:11201. doi: 10.15766/mep_2374-8265.11201 (PMC8720916; doi:10.15766/mep_2374-8265.11201)
Supplement: Supplementary file 1 — SP 1 Case.docxSP 1 Door Note.docxSP 2 Case.docxSP 2 Door Note.docxSP 3 Case.docxSP 3 Door Note.docxExample Interview Video.mp4Communication Rubric.docxReflection Prompts.docxFacilitators Guide.docx [file mep_2374-8265.11201-s001.zip › A. SP 1 Case.docx]

Appendix A: SP Case #1

Date: February 4 and 6, 2020

Primary Case Author: Beau Meyer

Secondary Case Author: Rocio Quinonez

Standardized Patient Educator: Bethany Fearnow

Name of Case: Infant Oral Health visit

Name of educational and or assessment activity: Formative assessment for motivational interviewing

Patient Name: Grayson

Chief Complaint: “Referred from pediatrician due to dark spots on front teeth.”

Most likely Diagnosis and Differential with rationale from history and/or physical exam: Early Childhood Caries (ECC), extrinsic staining

Challenge Question: “I’m sorry, but can we speed this up a little bit?”

Domains: Check all that apply

- Professionalism
  - Communication and Interpersonal skills
  - Medical History
  - Physical exam
  - Shared Decision Making
  - Patient Education
  - Clinical Reasoning
  - Documentation
  - Handoff
  - Presentation
  - Other:

Type and level of learner: Second year dental students

Case Objectives: please list specific objectives for each of the domains you have checked above:

By the end of this activity, learners will be able to:

1. Apply motivational interviewing to an infant oral health visit using the baby oral health program (bOHP) template;

2. Use motivational interviewing to provide oral health counseling during an infant oral health visit.

| SETTING: outpatient, in patient, ED, home, nursing home, rehab, group etc. | Outpatient dental clinic |
| --- | --- |
| PATIENT PROFILE: Information about the “patient” that helps select an SP and helps the learner get an understanding of them as a person. SP will know more information about the patient than learner will ever ask but allows SP to portray a fully developed patient personality. If none of the items below are particulars for the case please write “all may be used.” | |
| Age range | 20-30 years old (new parent to a 2yo and 6-month old) |
| Religious/spiritual background | All may be used |
| Sex (e.g., male, female, intersex, transwoman, transman) | All may be used |
| Sexual Orientation (e.g., heterosexual, lesbian, gay, bisexual, pansexual, queer, asexual) | All may be used |
| Gender expression (e.g., man, woman, gender queer) | All may be used |
| Race/ethnicity: | All may be used |
| Physical description (e.g., BMI, height range) | All may be used (child is 25 pounds, average height) |
| Physical limitations | All may be used |
| Patient appearance (e.g., disheveled, hospital gown, business casual, casual) | Casual |
| Moulage + location (e.g., none, bruises, scars, body piercing, tattoos) | None |
| Affect (e.g., pleasant, cooperative) | Pleasant, caregiver is anxious (child is active and high energy) |
| Family group (e.g., who is family, who they live with) | Lives with spouse, 2-year old child, and 6-month old child |
| Education | Spouse 1—High school diploma  Spouse 2—High school diploma  2-year old child—attends daycare full time |
| Level of health literacy | Average |
| Employment, if any - present and past, noting any current stresses | Spouse 1—bank teller  Spouse 2—construction worker |
| Home/homeless - type of dwelling, number of stories, owned or rented | Standalone, single family home |
| Financial situation- any current stresses | All may be used |
| Insurance Status (e.g., un/under/insured, public/private, HMO/PPO) | Private medical insurance through Spouse 1’s employer; no dental benefit |
| Habits (i.e., diet, exercise, caffeine, smoking, alcohol, drugs) | Diet (for child):   - Average 2 year old diet—mix of healthy and unhealthy foods - Milk and juice at daycare - Enjoys snacking (chips, cheezits, goldfish crackers)   Oral Hygiene (for child):   - Brushes his teeth only when he’ll allow it: “Not worth the fight” - When he does brush, he uses a non-fluoridated toothpaste - Only drinks bottled water—family doesn’t like the taste of tap water |
| Activities (i.e., hobbies, sports, clubs, friends) | For child: Active child, loves to play outside |
| Typical day - what is the usual daily routine | For SP: Takes kids to daycare at 7am, goes to work from 7:30am to 4pm. Picks up child from grandmother at 5pm.  For child:  Daycare full time, grandmother takes care of him between 2:30pm and 5:00pm  Bedtime routine: Brushes his teeth, reads a book, gets bottle of milk, bedtime is around 9pm |

| CASE INFORMATION | |
| --- | --- |
| Chief Concern: What the patient will say when greeted by the student. The patient’s primary reason for seeking medical care often stated in his/own words. | Your child has been referred from pediatrician due to dark spots on front teeth. |
| Additional Concerns: Other, if any, concerns the patient has today (i.e., symptoms, requests, expectations, etc.) that will become part of set agenda. | The SP would like to figure out  1) if my child has cavities,  2) if he does, how did he get them, and  3) do we have to fix them |
|  | |
| THE PATIENT STORY: The SP will be asked to tell their symptom story and the personal and emotion impact for each of their concerns. You will want to write this is the patient voice. The symptom story should be able to answer this question: “Tell me more about [chief concern/additional concern], starting at the beginning and bringing me up to now.”  The personal context should be able to answer questions concerning the broader personal/psychosocial context of symptoms, especially the patient beliefs/attributions.  The emotional context should be able to ask how are you doing with this, how does this make you feel, how has this affected you emotionally? IMPACT: How has this affected your life? How has this been for your family? | We’ve been going to our well child visits on schedule for two years now, and within the last 2 or 3 visits, the pediatrician started doing oral screenings. They asked to do fluoride varnish, but I declined. I didn’t think kids needed fluoride until they turned 3 years old.  At his last visit, the doctor noticed a few dark spots on the front teeth, and he referred us to you.  I’m a little uncertain with why we needed to come in today. These are just baby teeth, and they’re going to fall out someday. But I also feel a little bit guilty if these are cavities because I don’t want my baby to need fillings or to lose his teeth too early. I go to the dentist regularly, but my husband does not. He’s so afraid of the dentist because he had a terrible dental experience when he was growing up.  I didn’t know kids this young needed to go to the dentist. |
| HISTORY OF PRESENT ILLNESS: Although some of the HPI will be given in the patient’s symptom story, the learners will expand the story during the direct question section. Below describe the detailed history, usually about the chief concern, which the student must develop in order to make a useful assessment of the problem: | |
|  | |
| Onset (when; gradual or sudden) | No pain or dental sensitivity  Denies swelling or difficulty opening mouth fully  Unsure when dark spots started |
| Setting (what was going on or where was patient when symptoms first noticed?) | The pediatrician pointed out the spots at his last well child visit |
| Duration (how long) | That was 3 months ago |
| Time relationships (frequency, constant or intermittent) | n/a |
| Location | Front 4 teeth on the top |
| Radiation | n/a |
| Quality | n/a |
| Amount | n/a |
| Aggravated by what | n/a |
| Relieved by what | n/a |
| Associated with what | n/a |
| Attitude (what does the patient think is the problem, and how does he/she feel about it) | Are these cavities? How did he get them? Do we need to fix them? I didn’t know children needed to go to the dentist at this age. |
| Overall course | New finding, asymptomatic |
| REVIEW OF SYSTEMS: Significant positives and negatives | |
| Positives | n/a |
| Negatives | n/a |
| Past medical history |  |
| Medication allergies (Name and reaction) | Amoxicillin—he gets a rash on his chest |
| Environmental allergies (Name and reaction) | No known allergies |
| Illnesses | Frequent ear infections earlier in life |
| Vaccinations | Up to date for his age |
| Surgeries | Had ear tubes placed 6 months ago |
| Accidents/ injuries/ trauma | He fell on his face once when he was learning to walk but did not have any significant injury |
| Hospitalization | n/a |
|  | |
| Inclusive sexual and reproductive history | |
| Sexual practices  Sexual partners  Protection: Use of safer sex practices  Use of birth control if appropriate  Risk of intimate partner violence | n/a |
| Medications | OTC Tylenol as needed for pain or fever control |
| Immunizations | Up to date on all vaccines for his age   - Tetanus - Flu - Hepatitis - Pneumovax - HPV - Other |
| Tobacco products:   - Cigarettes - Cigar - Pipe - Chew - E-cigarettes | - Never - Past- year started/year quit - Current   - Quantity   - # of years |
| Alcohol   - Beer - Wine - Liquor - Other | - Never - Past- year started/year quit - Current   - Quantity   - # of years |
| Drugs   - Weed - Cocaine - Heroin - Meth - Other - IV - Inhalants - Other | - Never - Past- year started/year quit - Current   - Quantity - # of years |
| Diet (describe) | Diet:   - Average 2 year old diet—mix of healthy and unhealthy foods - Milk and juice at daycare - Enjoys snacking (chips, cheezits, goldfish crackers) |
| Exercise (describe) | Very active, loves playing outside |
| List any other important social history or information important to this case | n/a |
| Family history |  |
| Mother, Father, Siblings, Grandparents, and other significant findings. | Mother goes to the dentist every 6 months  Father does not go to the dentist regularly |
|  |  |
| Physical Exam- List exam maneuvers expected for this case and any abnormal findings that SP will simulate. (tenderness, hyper-hypo reflex, rebound, weakness etc. )  *Currently, unable to conduct an oral exam on a child patient actor at our institution. Instead, given clinical photos.* | |
| PHYSICAL EXAM FINDINGS |  |
| 1. Written in layman’s terms | Cavities on the front 4 teeth |
| 1. General appearance- affect, appearance, position of patient at opening (i.e. sitting, laying down, holding abdomen etc.) | Anxious about the dental visit; mom didn’t know he was supposed to have a dental visit before age 3 |
| 1. Vital signs | Unable to obtain |
| 1. Specific findings and affect | Hyper child, unable to sit still, resistant to directions when things do not go his way |
| 1. Response to certain physical movements | If child actor available, cries when reclining for a knee-to-knee exam |
|  |  |
| DIAGNOSIS AND DIFFERENTIAL |  |
| Diagnosis with support from positive and negative history and PE findings | Early childhood caries |
| Differential with support from positive and negative history and PE findings | Extrinsic staining |
|  |  |
| MANAGEMENT OR DIAGNOSTIC PLAN | Oral health counseling, scheduling return visits |
|  |  |
| PROFESSIONALISM ISSUES OR CHALLENGES: | Communicating disease diagnosis and rationale/importance of treatment |
