# Supplementary material for: Implementing Standardized Patient Caregivers to Practice Difficult Conversations in a Pediatric Dentistry Course
Source: MedEdPORTAL. 2022 Jan 3;18:11201. doi: 10.15766/mep_2374-8265.11201 (PMC8720916; doi:10.15766/mep_2374-8265.11201)
Supplement: Supplementary file 1 — SP 1 Case.docxSP 1 Door Note.docxSP 2 Case.docxSP 2 Door Note.docxSP 3 Case.docxSP 3 Door Note.docxExample Interview Video.mp4Communication Rubric.docxReflection Prompts.docxFacilitators Guide.docx [file mep_2374-8265.11201-s001.zip › B. SP 1 Door Note.docx]

**Patient Name: Grayson**

**Patient Age: 2 years old**

Patient has been referred by the pediatrician to the Adams School of Dentistry Pediatric clinic due to possible caries on #D, #E, #F, #G.

Please introduce yourself to the caregiver. Complete an infant oral health visit up to the clinical exam. Using motivational interviewing techniques, be sure to discuss CC/reason for visit/MedHx/Family Hx (5 minutes) and provide oral health counseling regarding diet/hygiene/fluoride exposure/habits/trauma (10 minutes) as part of a bOHP exam.

When you have finished, you will exit the Exam Room and you may then leave.

The course director will send you instructions later today to complete a reflection on the experience and your performance.
