# Supplementary material for: Implementing Standardized Patient Caregivers to Practice Difficult Conversations in a Pediatric Dentistry Course
Source: MedEdPORTAL. 2022 Jan 3;18:11201. doi: 10.15766/mep_2374-8265.11201 (PMC8720916; doi:10.15766/mep_2374-8265.11201)
Supplement: Supplementary file 1 — SP 1 Case.docxSP 1 Door Note.docxSP 2 Case.docxSP 2 Door Note.docxSP 3 Case.docxSP 3 Door Note.docxExample Interview Video.mp4Communication Rubric.docxReflection Prompts.docxFacilitators Guide.docx [file mep_2374-8265.11201-s001.zip › D. SP 2 Door Note.docx]

**Patient Name: Taylor**

**Patient Age: 13 years old**

Patient has been referred to the Adams School of Dentistry Pediatric Clinic from private practice due to loss of insurance.

Navigate difficult conversations in the context of a preventive oral health visit (CC/reason for visit/MedHx/Family Hx/Social Hx) with parents of an adolescent dental patient (7-8 minutes).

Provide individualized oral health counseling to an adolescent patient from a vulnerable population using the intraoral photo below (7-8 minutes).

When you have finished, you will exit the Exam Room and you may then leave.

The course director will send you instructions later today to complete a reflection on the experience and your performance.


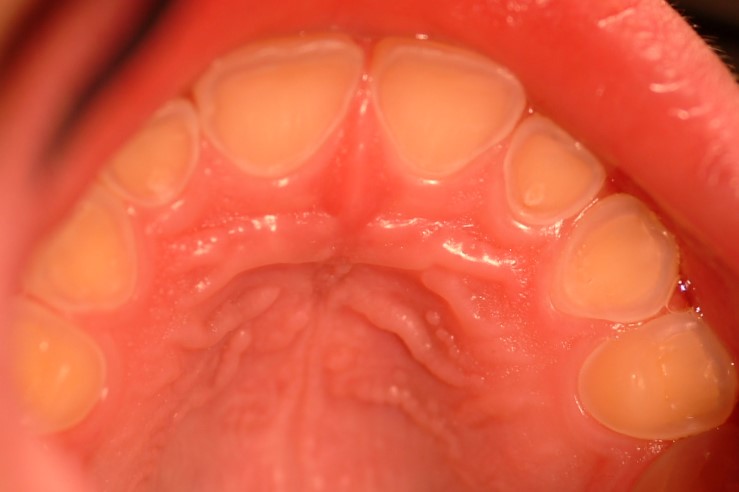


“Image by University of North Carolina at Chapel Hill, Adams School of Dentistry, Division of Diagnostic Sciences, used with permission.”
