# Supplementary material for: Implementing Standardized Patient Caregivers to Practice Difficult Conversations in a Pediatric Dentistry Course
Source: MedEdPORTAL. 2022 Jan 3;18:11201. doi: 10.15766/mep_2374-8265.11201 (PMC8720916; doi:10.15766/mep_2374-8265.11201)
Supplement: Supplementary file 1 — SP 1 Case.docxSP 1 Door Note.docxSP 2 Case.docxSP 2 Door Note.docxSP 3 Case.docxSP 3 Door Note.docxExample Interview Video.mp4Communication Rubric.docxReflection Prompts.docxFacilitators Guide.docx [file mep_2374-8265.11201-s001.zip › E. SP 3 Case.docx]

Appendix E: SP Case #3

Date: March 31, 2020

Primary Case Author: Beau Meyer

Secondary Case Author: Rocio Quinonez

Standardized Patient Educator: Bethany Fearnow

Name of Case: Pediatric Dental Treatment Planning

Name of educational and or assessment activity: Summative assessment for counseling and treatment planning

Patient Name: Zach

Chief Complaint: “Referred here for cavities due to behavior.”

Most likely Diagnosis and Differential with rationale from history and/or physical exam: Dental caries with isolated instances of pulpally involved teeth

Non-odontogenic sources of pain

Erupting teeth

Challenge question: “I hear what you are saying, but does he really need all of that metal in his mouth? And why the fluoride? I don’t see the benefit.”

Domains: Check all that apply

- Professionalism
  - Communication and Interpersonal skills
  - Medical History
  - Physical exam
  - Shared Decision Making
  - Patient Education
  - Clinical Reasoning
  - Documentation
  - Handoff
  - Presentation
  - Other:

Type and level of learner: Second year dental students

Case Objectives: please list specific objectives for each of the domains you have checked above:

By the end of this educational activity, the learner will be able to:

1. Generate pediatric dental treatment options based on the caregiver interview and clinical information; and

2. Navigate difficult conversations in the context of a preventive oral health visit with parents of a pediatric dental patient

| SETTING: outpatient, in patient, ED, home, nursing home, rehab, group etc. | Outpatient dental clinic |
| --- | --- |
| PATIENT PROFILE: Information about the “patient” that helps select an SP and helps the learner get an understanding of them as a person. SP will know more information about the patient than learner will ever ask but allows SP to portray a fully developed patient personality. If none of the items below are particulars for the case please write “all may be used.” | |
| Age range | 30-40 years old (Child is 6 years old) |
| Religious/spiritual background | All may be used |
| Sex (e.g., male, female, intersex, transwoman, transman) | All may be used (child is male) |
| Sexual Orientation (e.g., heterosexual, lesbian, gay, bisexual, pansexual, queer, asexual) | All may be used |
| Gender expression (e.g., man, woman, gender queer) | All may be used |
| Race/ethnicity: | All may be used |
| Physical description (e.g., BMI, height range) | 50^th^ percentile weight and height |
| Physical limitations | None |
| Patient appearance (e.g., disheveled, hospital gown, business casual, casual) | Casual |
| Moulage + location (e.g., none, bruises, scars, body piercing, tattoos) | None |
| Affect (e.g., pleasant, cooperative) | Pleasant |
| Family group (e.g., who is family, who they live with) | Spouse no longer in the child’s life  Single sibling—12 years old  Child lives with SP (can be mother or father) |
| Education | Child attends public school  Parent has a high school education |
| Level of health literacy | Average |
| Employment, if any - present and past, noting any current stresses | SP is a firefighter |
| Home/homeless - type of dwelling, number of stories, owned or rented | Lives in a condo complex |
| Financial situation- any current stresses | All may be used |
| Insurance Status (e.g., un/under/insured, public/private, HMO/PPO) | Medical and dental insurance through parent’s employer |
| Habits (i.e., diet, exercise, caffeine, smoking, alcohol, drugs) | For Child:  Diet   - Hot dogs or chicken nuggets, Cheetos - No vegetables - Favorite drink: Soda/sugar sweetened, carbonated beverages - Does not like the taste of water   Oral Hygiene   - Only brushes when told to, and even then it is just “a quick scrub” - When he does brush, it is with a non-fluoridated toothpaste - No flossing or mouth rinse |
| Activities (i.e., hobbies, sports, clubs, friends) | For child:  Doesn’t like playing outside  Loves trains |
| Typical day - what is the usual daily routine | SP: Helps get child ready for day and takes to school at 7:30am. Works three 12-hour days in a row, then has three days off, and the cycle repeats.  For child:  Wake up, 30 minutes of play, breakfast, school  Return from school, 2 hours of play, dinner, 2 hours of videos, bed  Bedtime routine: Reads a book, drinks a glass of warm milk, then goes to bed; toothbrushing is a struggle |

| CASE INFORMATION | |
| --- | --- |
| Chief Concern: What the patient will say when greeted by the student. The patient’s primary reason for seeking medical care often stated in his/own words. | “He was referred for cavities due to behavior.” |
| Additional Concerns: Other, if any, concerns the patient has today (i.e., symptoms, requests, expectations, etc.) that will become part of set agenda. | I want to know why he has cavities and what the plan is to fix them. Next, I am also hesitant to use fluoride because I don’t want it to make Zach’s autism worse. If we decide to use it, and that’s a big if, I want to know how it works, how much to use, and how often it should be used Last, I hear sometimes kids get put to sleep to fix teeth. This makes me really nervous because my mom had an extended hospital stay following what was described as a routine knee procedure. She experienced an adverse cardiac event while under anesthesia. |
|  | |
| THE PATIENT STORY: The SP will be asked to tell their symptom story and the personal and emotion impact for each of their concerns. You will want to write this is the patient voice. The symptom story should be able to answer this question: “Tell me more about [chief concern/additional concern], starting at the beginning and bringing me up to now.”  The personal context should be able to answer questions concerning the broader personal/psychosocial context of symptoms, especially the patient beliefs/attributions.  The emotional context should be able to ask how are you doing with this, how does this make you feel, how has this affected you emotionally? IMPACT: How has this affected your life? How has this been for your family? | We’ve had a really hard time with Zach and the dentist. We tried that whole desensitization thing at the last dentist starting when Zach was 3 years old, but it never worked. Shortly after we started these types of visits, my spouse left the family. So it’s just me, and honestly, dental care took a backseat to figuring out this new life because Zach hates going to the dentist.  About a month ago, Zach started holding his mouth at random times, but I could never figure out why. Now I’m thinking it’s because his teeth might be bothering him. It’s never caused him to change his routine. So I took him back to his last dentist, and somehow the dentist was able to get a good enough look to know he has cavities. However, he had to send us to you because he couldn’t manage Zach’s behavior.  We don’t use fluoride in our house because of Zach’s autism. I hear that stuff makes the autism worse. We also don’t like any metals for the same reason. I’m curious what you have to say about his cavities and how you propose we fix them. |
| HISTORY OF PRESENT ILLNESS: Although some of the HPI will be given in the patient’s symptom story, the learners will expand the story during the direct question section. Below describe the detailed history, usually about the chief concern, which the student must develop in order to make a useful assessment of the problem: | |
|  | |
| Onset (when; gradual or sudden) | Possible tooth pain for about a month, random onsets |
| Setting (what was going on or where was patient when symptoms first noticed?) | Unsure, no particular issue that seemed to cause him to reach up and grab his mouth. |
| Duration (how long) | It lasts for about 30 minutes and either goes away or he stops caring about it |
| Time relationships (frequency, constant or intermittent) | Intermittent |
| Location | Generalized to the mouth, no specific area |
| Radiation | n/a |
| Quality | Unsure—he can’t really communicate any descriptive information |
| Amount | Unsure |
| Aggravated by what | Unsure |
| Relieved by what | Unsure |
| Associated with what | Unsure |
| Attitude (what does the patient think is the problem, and how does he/she feel about it) | Unsure—he can’t really communicate any descriptive information |
| Overall course | Unsure—he can’t really communicate any descriptive information |
| REVIEW OF SYSTEMS: Significant positives and negatives | |
| Positives | n/a |
| Negatives | n/a |
| Past medical history | For child:  Autism spectrum disorder (low functioning, minimal language)  ADHD |
| Medication allergies (Name and reaction) | No known medication allergies |
| Environmental allergies (Name and reaction) | For child:  Peanut butter |
| Illnesses | None |
| Vaccinations | SP is anti-vaccinations |
| Surgeries | Child had tonsils and adenoids removed at 3 years old |
| Accidents/ injuries/ trauma | None |
| Hospitalization | None |
|  | |
| Inclusive sexual and reproductive history | |
| Sexual practices  Sexual partners  Protection: Use of safer sex practices  Use of birth control if appropriate  Risk of intimate partner violence | n/a |
| Medications | Used to take focalin daily, but it wasn’t really working so I stopped giving it to him |
| Immunizations | None—we don’t vaccinate Zach because we don’t want to make his autism worse   - Tetanus - Flu - Hepatitis - Pneumovax - HPV - Other |
| Tobacco products:   - Cigarettes - Cigar - Pipe - Chew - E-cigarettes | - Never - Past- year started/year quit - Current   - Quantity   - # of years |
| Alcohol   - Beer - Wine - Liquor - Other | - Never - Past- year started/year quit - Current   - Quantity   - # of years |
| Drugs   - Weed - Cocaine - Heroin - Meth - Other - IV - Inhalants - Other | - Never - Past- year started/year quit - Current   - Quantity - # of years |
| Diet (describe) | For child:  Hot dogs or chicken nuggets, Cheetos  No vegetables  Favorite drink: Soda/sugar sweetened, carbonated beverages  Does not like the taste of water |
| Exercise (describe) | Child doesn’t get much outdoor play, mostly plays inside |
| List any other important social history or information important to this case | n/a |
| Family history |  |
| Mother, Father, Siblings, Grandparents, and other significant findings. | All may be used |
|  |  |
| Physical Exam- List exam maneuvers expected for this case and any abnormal findings that SP will simulate. (tenderness, hyper-hypo reflex, rebound, weakness etc.)  *Currently, unable to conduct an oral exam on a child patient actor at our institution. Instead, given clinical photos.* | |
| PHYSICAL EXAM FINDINGS |  |
| 1. Written in layman’s terms | Generalized dental cavities |
| 1. General appearance- affect, appearance, position of patient at opening (i.e. sitting, laying down, holding abdomen etc.) | Sitting in the exam room, interactive, curious to learn more and discuss a plan of care |
| 1. Vital signs | Unable to obtain on Zach |
| 1. Specific findings and affect | Zach is pacing back and forth in the exam room, and extremely afraid of the dentist |
| 1. Response to certain physical movements | If child actor available, becomes hyper and belligerent during a knee-to-knee exam |
|  |  |
| DIAGNOSIS AND DIFFERENTIAL |  |
| Diagnosis with support from positive and negative history and PE findings | Generalized and severe dental caries, with isolated areas of pulpally involved teeth |
| Differential with support from positive and negative history and PE findings | Non-odontogenic source of pain  Erupting teeth |
|  |  |
| MANAGEMENT OR DIAGNOSTIC PLAN | Present a menu of options; due to extent and severity of disease, as well as Zach’s behavior, he will likely require general anesthesia to complete dental treatment |
|  |  |
| PROFESSIONALISM ISSUES OR CHALLENGES: | Presenting the best available evidence in an unbiased way that is both sensitive to the emotions/opinions of the parent and provides Zach with the best chance of improving his quality of life |
