# Supplementary material for: Implementing Standardized Patient Caregivers to Practice Difficult Conversations in a Pediatric Dentistry Course
Source: MedEdPORTAL. 2022 Jan 3;18:11201. doi: 10.15766/mep_2374-8265.11201 (PMC8720916; doi:10.15766/mep_2374-8265.11201)
Supplement: Supplementary file 1 — SP 1 Case.docxSP 1 Door Note.docxSP 2 Case.docxSP 2 Door Note.docxSP 3 Case.docxSP 3 Door Note.docxExample Interview Video.mp4Communication Rubric.docxReflection Prompts.docxFacilitators Guide.docx [file mep_2374-8265.11201-s001.zip › F. SP 3 Door Note.docx]

**Patient Name: Zach**

**Patient Age: 6 years old**

Patient has been referred to the Adams School of Dentistry Pediatric Clinic for cavities due to behavior.

Review the recorded SP interview posted to the course website. First, respond to the caregiver questions about Zach’s teeth. (3-5 minutes). Second, generate a list of behavior management and disease management options based on the interview and the clinical information shown below (10-12 minutes).

The course director will send you instructions later today to complete a reflection on the experience and your performance.


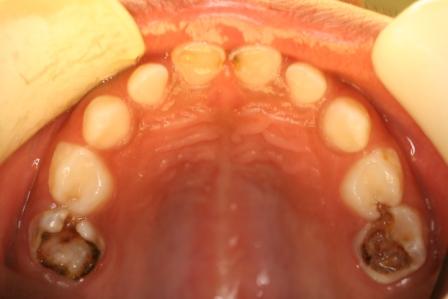


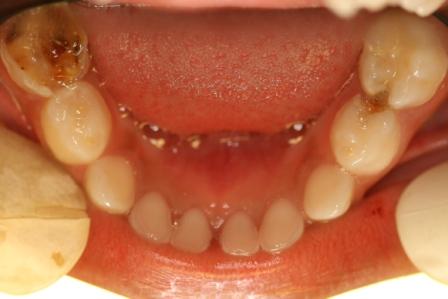


“Image by University of North Carolina at Chapel Hill, Adams School of Dentistry, Division of Pediatric and Public Health, used with permission.”
