# Supplementary material for: Implementing Standardized Patient Caregivers to Practice Difficult Conversations in a Pediatric Dentistry Course
Source: MedEdPORTAL. 2022 Jan 3;18:11201. doi: 10.15766/mep_2374-8265.11201 (PMC8720916; doi:10.15766/mep_2374-8265.11201)
Supplement: Supplementary file 1 — SP 1 Case.docxSP 1 Door Note.docxSP 2 Case.docxSP 2 Door Note.docxSP 3 Case.docxSP 3 Door Note.docxExample Interview Video.mp4Communication Rubric.docxReflection Prompts.docxFacilitators Guide.docx [file mep_2374-8265.11201-s001.zip › H. Communication Rubric.docx]

| Step 1: Sets the Stage | | | |
| --- | --- | --- | --- |
| Behavior | Explanation | Y/N | Notes |
| 1. **Welcomes the patient** | Greets the patient warmly and shakes hands if appropriate |  |  |
| 2. **Addresses the patient** | Uses the name the patient prefers to be called. |  |  |
| 3. **Introduces themselves** | Identifies themselves as a student member of the health care team and explains their role. |  |  |
| 4. **Ensures patient readiness** **and privacy** | Closes doors or curtains, verifies identities of any third parties, and discretely excuses third parties as indicated by situation or patient preference. |  |  |
| 5. **Removes barriers** **to communication** | Addresses any physical, emotional, or environmental circumstances that may preclude an effective interaction. |  |  |
| 6. **Ensures comfort** **and puts the patient at ease** | Speaks to the patient kindly and politely before beginning the interview. This may include light conversation as needed. |  |  |
| Example of Step 1: “Good afternoon, Mrs. Green. My name is John Smith, I’m the medical student on your team. It’s nice to meet you (closes the door to ensure privacy; shakes patient’s hand; positions chair so that he is at the same eye level with the patient). Thank you for coming in today. “ | | | |
|  | | | |
| Step 2: Obtains the Chief Complaint and Sets the Agenda | | | |
| Behavior | Explanation | Y/N | Notes |
| 7. **Obtains the chief complaint** | Uses an open-ended question to elicit the chief complaint from the patient in their words. |  |  |
| 8. **Obtains a list of any other issues the patient wants to discuss** | Acknowledges importance of chief complaint and asks “Is there anything else you want to discuss or accomplish today?”. |  |  |
| Example of Step 2: “How can I help you this afternoon? I’m sorry you’re having headaches. They can be really frustrating. Before we discuss those headaches, is there anything else you want to make sure we accomplish or discuss this afternoon?” | | | |

| Step 3: Explores the History of the Present Illness | | | | | | | | | | |
| --- | --- | --- | --- | --- | --- | --- | --- | --- | --- | --- |
| Behavior | | Explanation | | Y/N | | | Notes | | | |
| 9. **Invites elaboration of the chief complaint** | | Uses an open-ended phrase or question to invite further description of the chief complaint. (2 pts) | |  | | |  | | | |
| 10. **Listens attentively and invites patient to talk freely** | | Remains silent and uses non-verbal cues to encourage patient to talk. May follow up with another open-ended question. (2 pts) | |  | | |  | | | |
| 11. **Summarizes the HPI and invites corrections**(1 pt) | | Reviews the patient’s description of their symptoms and encourages patient correction or errors | |  | | |  | | | |
| Example of Step 3: “Okay, tell me about these headaches.” (Remains silent while the patient talks; nods head, leans forward, intermittently nods to encourage patient to keep talking.) “Thank you. That’s helpful. What else can you tell me about them?” | | | | | | | | | | |
|  | | | | | | | | | | |
| Step 4: Uses Facilitation Skills Throughout the Interview | | | | | | | | | | |
| Behavior | | | Explanation | | Y/N | | | Notes | |  |
| 12. **Empathy** | | | Displays empathy through verbal responses to patient’s expressed emotions or situation.  Ex: “I’m so sorry to hear that.” | |  | | |  | |  |
| 13. **Understanding** | | | Indicates comprehension of a patient’s situation or emotion.  Ex: “I understand. Thank you for sharing that with me.” | |  | | |  | |  |
| 14. **Respect** | | | Praises the patient or acknowledges the patient’s situation.  Ex: “You’re really dealing with a lot right now. “ | |  | | |  | |  |
| 15. **Nonverbal encouragement** | | | Encourages the patient to speak with judicious use of gestures, eye contact, or body language to encourage patient discussion.  Ex: Leaning in, nodding, maintaining eye contact, “yes, uh-huh” | | |  | | |  |  |
|  | | | | | | | | | | |
|  | | | | | | | | | | |
|  | | | | | | | | | | |
|  | | | | | | | | | | |
| Step 5: Concludes the Interview | | | | | | | | | | |
| Behavior | Explanation | | | Y/N | | | Notes | | | |
| 16. **Invites Questions** | Encourages the patient to ask questions.  Ex: “Thank you for answering all of my questions today. Do you have any questions for me?” | | |  | | |  | | | |
| 17. **Explores patient’s concerns** | Seeks to identify any worries or fears the patient may have about the current symptoms.  Ex: “The information you’ve given me is really helpful. We’re going to do our best to figure this out. As we do so, is there anything in particular you’re worried this could be? I want to make sure we address those concerns.” | | |  | | |  | | | |
| 18. **Concludes and explains next steps** | Explains they will be leaving to discuss the case with their supervising physician and will return shortly to talk about the treatment plan.  Ex: “Okay, I think I have everything I need. I’m going to step out now to review this information with my supervising physician. I’ll be back in just a few minutes to share our thoughts and talk about the treatment plan.” | | |  | | |  | | | |
