# Supplementary material for: Implementing Standardized Patient Caregivers to Practice Difficult Conversations in a Pediatric Dentistry Course
Source: MedEdPORTAL. 2022 Jan 3;18:11201. doi: 10.15766/mep_2374-8265.11201 (PMC8720916; doi:10.15766/mep_2374-8265.11201)
Supplement: Supplementary file 1 — SP 1 Case.docxSP 1 Door Note.docxSP 2 Case.docxSP 2 Door Note.docxSP 3 Case.docxSP 3 Door Note.docxExample Interview Video.mp4Communication Rubric.docxReflection Prompts.docxFacilitators Guide.docx [file mep_2374-8265.11201-s001.zip › I. Reflection Prompts.docx]

**Appendix I.** Peer-to-Peer Reflection Prompts

1. Describe two things that went well with this encounter.
2. What motivational interviewing prompts did you use? Which ones would you use differently next time?
3. Describe two things that were challenging for you during this encounter.
4. List two suggestions that we can use to improve the next encounter.
