# Supplementary material for: Implementing Standardized Patient Caregivers to Practice Difficult Conversations in a Pediatric Dentistry Course
Source: MedEdPORTAL. 2022 Jan 3;18:11201. doi: 10.15766/mep_2374-8265.11201 (PMC8720916; doi:10.15766/mep_2374-8265.11201)
Supplement: Supplementary file 1 — SP 1 Case.docxSP 1 Door Note.docxSP 2 Case.docxSP 2 Door Note.docxSP 3 Case.docxSP 3 Door Note.docxExample Interview Video.mp4Communication Rubric.docxReflection Prompts.docxFacilitators Guide.docx [file mep_2374-8265.11201-s001.zip › C. SP 2 Case.docx]

Appendix E: SP Case #2

Date: February 25, 2020

Primary Case Author: Beau Meyer

Secondary Case Author: Rocio Quinonez

Standardized Patient Educator: Bethany Fearnow

Name of Case: Adolescent oral health visit

Name of educational and or assessment activity: Formative assessment for oral health counseling

Patient Name: Taylor

Chief Complaint: “Referred from private practice to the dental school due to loss of insurance.”

Most likely Diagnosis and Differential with rationale from history and/or physical exam:

Dental erosion subsequent to bulimia

Dental erosion subsequent to GERD

Dental attrition

Challenge Question: “I can’t believe she’s doing this; never thought we’d be here discussing this. Is this just all in her head?”

Domains: Check all that apply

- Professionalism
  - Communication and Interpersonal skills
- Medical History
  - Physical exam
  - Shared Decision Making
  - Patient Education
  - Clinical Reasoning
  - Documentation
  - Handoff
  - Presentation
  - Other:

Type and level of learner: Second year dental student

Case Objectives: please list specific objectives for each of the domains you have checked above:

By the end of this activity, learners will be able to:

1. Provide individualized oral health counseling to an adolescent patient from a vulnerable population; and

2. Navigate difficult conversations in the context of a preventive oral health visit with parents of an adolescent dental patient.

| SETTING: outpatient, in patient, ED, home, nursing home, rehab, group etc. | Outpatient dental clinic |
| --- | --- |
| PATIENT PROFILE: Information about the “patient” that helps select an SP and helps the learner get an understanding of them as a person. SP will know more information about the patient than learner will ever ask but allows SP to portray a fully developed patient personality. If none of the items below are particulars for the case please write “all may be used.” | |
| Age range | 30-40 years old (Child is 13 years old) |
| Religious/spiritual background | All may be used |
| Sex assigned at birth (e.g., male, female, intersex, transwoman, transman) | All may be used (Child is male) |
| Sexual Orientation (e.g., heterosexual, lesbian, gay, bisexual, pansexual, queer, asexual) | All may be used (Child identifies as queer) |
| Gender expression (e.g., masculine, feminine, an) | All may be used (Child is feminine (pronouns she, her, hers)) |
| Race/ethnicity: | All may be used |
| Physical description (e.g., BMI, height range) | All may be used (Child is underweight for age, normal height) |
| Physical limitations | All may be used |
| Patient appearance (e.g., disheveled, hospital gown, business casual, casual) | Casual |
| Moulage + location (e.g., none, bruises, scars, body piercing, tattoos) | None |
| Affect (e.g., pleasant, cooperative) | Pleasant, quiet and reserved (child is also quiet and reserved) |
| Family group (e.g., who is family, who they live with) | Child lives with SP  SP divorced from spouse two years ago due to disagreements about child’s gender affirmation  No siblings |
| Education | Child—attends high school  Spouse 1 (SP)—Bachelor’s degree  Spouse 2—Bachelor’s degree |
| Level of health literacy | High |
| Employment, if any - present and past, noting any current stresses | Spouse 1 (SP)—manages a daycare  Spouse 2—car salesman |
| Home/homeless - type of dwelling, number of stories, owned or rented | Lives on the second story of a three story apartment building in town |
| Financial situation- any current stresses | All may be used |
| Insurance Status (e.g., un/under/insured, public/private, HMO/PPO) | Private medical insurance, but recently lost dental insurance |
| Habits (i.e., diet, exercise, caffeine, smoking, alcohol, drugs) | For the child:  Diet:   - Lots of fruits and vegetables, little meat or dairy, limited sweets - Binges and purges   Oral Hygiene:   - Brushes teeth always in the morning, only sometimes in the evening - Uses fluoride toothpaste - Does not floss or use a mouth rinse |
| Activities (i.e., hobbies, sports, clubs, friends) | All may be used |
| Typical day - what is the usual daily routine | For the child:  Wakes up at 5:00am to exercise before school  Exercises for 2 hours after school  No regular extracurricular activities |

| CASE INFORMATION | |
| --- | --- |
| Chief Concern: What the patient will say when greeted by the student. The patient’s primary reason for seeking medical care often stated in his/own words. | Our former dentist referred us here because we lost our dental insurance. We are looking to establish care here. |
| Additional Concerns: Other, if any, concerns the patient has today (i.e., symptoms, requests, expectations, etc.) that will become part of set agenda. | I’d like to know if her gender-affirming hormone therapy is going to affect her oral health. Is there anything I need to look out for?  I’d also like to know more about what the eating disorder will do to her teeth. |
|  | |
| THE PATIENT STORY: The SP will be asked to tell their symptom story and the personal and emotion impact for each of their concerns. You will want to write this is the patient voice. The symptom story should be able to answer this question: “Tell me more about [chief concern/additional concern], starting at the beginning and bringing me up to now.”  The personal context should be able to answer questions concerning the broader personal/psychosocial context of symptoms, especially the patient beliefs/attributions.  The emotional context should be able to ask how are you doing with this, how does this make you feel, how has this affected you emotionally? IMPACT: How has this affected your life? How has this been for your family? | Taylor and I have been going to the dentist for routine checks for basically her whole life. About two years ago, she came out to her father and me when she said she didn’t feel like a boy. We were confused and in denial. Her father didn’t take this very well and couldn’t wrap his head around it. He started avoiding her and me, and eventually we got a divorce.  While I can’t say I fully understand the process, I love my child and want to support her and do what is best. We recently lost our dental insurance, and our previous dentist was nice enough to recommend this clinic.  About one year ago, Taylor started getting bullied in school because of her gender identity emergence. As a way to cope, she started exercising all the time because she thought it would make her more attractive. She began to withdraw around meal times, and eventually I had to take her to the hospital because she was losing so much weight. We learned, then, that she has bulimia. She gets counseling for that, but she still has episodes of binging and purging. These are often accompanied by mood swings, irritability, and isolation, but it’s hard for me to tell if those are related to puberty, the eating disorder, or the emergence of her gender identity. I really don’t know what to do next. |
| HISTORY OF PRESENT ILLNESS: Although some of the HPI will be given in the patient’s symptom story, the learners will expand the story during the direct question section. Below describe the detailed history, usually about the chief concern, which the student must develop in order to make a useful assessment of the problem: | |
|  | |
| Onset (when; gradual or sudden) | She reports some sensitivity to cold, that comes and goes quickly |
| Setting (what was going on or where was patient when symptoms first noticed?) | It only occurs when she drinks something with ice in it. |
| Duration (how long) | It lasts only a few minutes |
| Time relationships (frequency, constant or intermittent) | Intermittent, isolated to drinking things with ice |
| Location | No specific area |
| Radiation | n/a |
| Quality | n/a |
| Amount | n/a |
| Aggravated by what | Drinking something with ice |
| Relieved by what | Time |
| Associated with what | Drinking something with ice |
| Attitude (what does the patient think is the problem, and how does he/she feel about it) | She’s not sure, doesn’t really care, and generally avoids cold drinks when possible |
| Overall course | She self-manages it fairly well |
| REVIEW OF SYSTEMS: Significant positives and negatives | |
| Positives | n/a |
| Negatives | n/a |
| Past medical history | Patient diagnosed with bulimia 1 year ago |
| Medication allergies (Name and reaction) | No known medication allergies |
| Environmental allergies (Name and reaction) | No known environmental allergies |
| Illnesses | n/a |
| Vaccinations | Up to date |
| Surgeries | n/a |
| Accidents/ injuries/ trauma | Broke her arm when she was 5 years old |
| Hospitalization | Hospitalized for severe weight loss 12 months ago |
|  | |
| Inclusive sexual and reproductive history | |
| Sexual practices  Sexual partners  Protection: Use of safer sex practices  Use of birth control if appropriate  Risk of intimate partner violence | For child:  Denies any exploration of sexual practices  Denies sexual partners |
| Medications | Currently none  Child is interested in beginning gender-affirming hormone therapy |
| Immunizations | Up to date on all vaccinations for age.   - Tetanus - Flu - Hepatitis - Pneumovax - HPV - Other |
| Tobacco products:   - Cigarettes - Cigar - Pipe - Chew - E-cigarettes | - Never - Past- year started/year quit - Current   - Quantity   - # of years |
| Alcohol   - Beer - Wine - Liquor - Other | - Never - Past- year started/year quit - Current   - Quantity   - # of years |
| Drugs   - Weed - Cocaine - Heroin - Meth - Other - IV - Inhalants - Other | - Never - Past- year started/year quit - Current   - Quantity - # of years |
| Diet (describe) | Lots of fruits and vegetables, little meat or dairy, limited sweets  Binges and purges |
| Exercise (describe) | Exercises before school for an hour  Exercises after school for two hours |
| List any other important social history or information important to this case | n/a |
| Family history |  |
| Mother, Father, Siblings, Grandparents, and other significant findings. | n/a |
|  |  |
| Physical Exam- List exam maneuvers expected for this case and any abnormal findings that SP will simulate. (tenderness, hyper-hypo reflex, rebound, weakness etc.)  *Currently, unable to conduct an oral exam on a child patient actor at our institution. Instead, given clinical photos.* | |
| PHYSICAL EXAM FINDINGS |  |
| 1. Written in layman’s terms | Dental erosion on the lingual aspect of maxillary teeth |
| 1. General appearance- affect, appearance, position of patient at opening (i.e. sitting, laying down, holding abdomen etc.) | Quiet and reserved |
| 1. Vital signs | All may be used |
| 1. Specific findings and affect | SP is reserved, but open to receiving oral health advice |
| 1. Response to certain physical movements | If child actor available, follows all directions from student provider |
|  |  |
| DIAGNOSIS AND DIFFERENTIAL |  |
| Diagnosis with support from positive and negative history and PE findings | Dental erosion subsequent to bulimia |
| Differential with support from positive and negative history and PE findings | Dental erosion subsequent to GERD  Dental attrition |
|  |  |
| MANAGEMENT OR DIAGNOSTIC PLAN | Oral health counseling, scheduling return visits |
|  |  |
| PROFESSIONALISM ISSUES OR CHALLENGES: | Not everyone has an experience interacting with an individual or a caregiver of a child who is on the path to gender affirmation. Still today, transgendered individuals face discrimination from different angles. This encounter was designed to provide students with exposure to this population group in a simulated setting. |
