# Supplementary material for: Implementing Standardized Patient Caregivers to Practice Difficult Conversations in a Pediatric Dentistry Course
Source: MedEdPORTAL. 2022 Jan 3;18:11201. doi: 10.15766/mep_2374-8265.11201 (PMC8720916; doi:10.15766/mep_2374-8265.11201)
Supplement: Supplementary file 1 — SP 1 Case.docxSP 1 Door Note.docxSP 2 Case.docxSP 2 Door Note.docxSP 3 Case.docxSP 3 Door Note.docxExample Interview Video.mp4Communication Rubric.docxReflection Prompts.docxFacilitators Guide.docx [file mep_2374-8265.11201-s001.zip › J. Facilitators Guide.docx]

FACILITATOR’S GUIDE

The three standardized patient experiences (SPE) used here are part of a pediatric dentistry course that provides second-year dental students (class size approximately 80) the opportunity to practice speaking with pediatric patient caregivers about challenging topics. Prior to the encounters, students received lectures about oral health topics tailored to caregivers of specific age groups (1. Infants, 2. Adolescents, 3. Kindergarteners). In each lecture, motivational interviewing was used as a framework to deliver oral health counseling while being aware of how social, cultural, and family factors influence oral health behaviors. Each SPE was designed to reinforce student learning through practical application of motivational interviewing in a controlled setting.

Standardized patients were selected based on case descriptions and trained with a case file provided by the faculty facilitator and a standardized patient educator. Each session included a brief orientation to the SPE, the actual SPE, and debriefing and feedback. Based on scheduling limitations, twelve exam rooms and standard patients were required, and the 84 individual sessions occurred over a two and a half hour period (7:30am to 10:00am). On the day of the SPEs, students were provided instructions and the evaluation rubric during a 15-minute orientation. They then received the case information immediately prior to entering the exam room. Students completed a 15-minute, one-on-one encounter with the standardized patient according to instructions given in the orientation and case materials. Encounters were video-recorded and graded by a faculty facilitator using the rubric provided to students.

After each session, students received their completed rubric with written feedback for improvement from the faculty facilitator and the standardized patient. They also watched their recorded SPEs with a peer/colleague to write a brief reflection on their performance. The course director led a 20-30 minute debriefing session during the subsequent class session.

Necessary Resources for implementation include:

1. Access to standardized patient space and actors (common in health professional school settings)
2. Staff to facilitate encounter flow
3. Ability to video record or otherwise monitor learners for assessment and feedback
4. Adequate faculty members to evaluate learners
5. An orientation to prepare students for the SPEs
6. Case documents: SP case information, instructions for learners, and evaluation rubrics

The following goals and expectations were communicated to students and standardized patients for each SPE.

Overall Goals for the STUDENT:

· Listen attentively to patient with a calm demeanor

· Practice communication skills

· Complete a caregiver interview

· Provide patient-specific oral health counseling

THE STUDENT SHOULD:

· Introduce him/herself

· Offer to shake hands

· Not take an intellectualizing approach (cite dental statistics) in response to the patient

· Not minimize/trivialize/discount the patient’s experience

· Be calm, reassuring, empathetic (“We will be able to help your child”)

· Maintain frequent eye contact

· Present as self-confident

· Take notes

**SP 1 - Motivational interviewing on caries prevention for toddlers**

The following expectations were specific to this encounter:

THE STUDENT SHOULD:

- Review and counsel on diet and feeding practices
- Review and counsel on oral hygiene practices and fluoride exposure
- Discuss oral habits (e.g. thumb-sucking) and trauma prevention

**SP 2 – Adolescent with an eating disorder on the path to gender affirmation**

This encounter was intended to practice cultural sensitivity, discuss sensitive topics such as eating disorders, and recognize bulimia and advise a patient/parent accordingly. The following expectations were specific to this encounter:

THE STUDENT SHOULD:

- Use correct patient pronouns
- Review details of clinical findings (erosion of teeth due to bulimia)
- Review and provide preventive counseling

**SP 3 – Kindergartner with Autism spectrum disorder**

This encounter was intended to quickly synthesize clinical and interview information to deliver a pediatric dental treatment plan within the context of the child’s overall behavior and parental concerns regarding the use of fluoride. The following expectations were specific to this encounter:

THE STUDENT SHOULD:

- Review and counsel dietary behaviors to optimize oral health
- Review clinical findings and deliver detailed prevention plan
- Discuss risks and benefits of at least two different treatment options, one of which includes referral for treatment under general anesthesia
