# Supplementary material for: Implementing Standardized Patient Caregivers to Practice Difficult Conversations in a Pediatric Dentistry Course
Source: MedEdPORTAL. 2022 Jan 3;18:11201. doi: 10.15766/mep_2374-8265.11201 (PMC8720916; doi:10.15766/mep_2374-8265.11201)
Supplement: Supplementary file 1 — SP 1 Case.docxSP 1 Door Note.docxSP 2 Case.docxSP 2 Door Note.docxSP 3 Case.docxSP 3 Door Note.docxExample Interview Video.mp4Communication Rubric.docxReflection Prompts.docxFacilitators Guide.docx [file mep_2374-8265.11201-s001.zip › Educational_Summary_Report_11201.pdf]

MedEdPORTAL® The AAMC Journal of Teaching and Learning Resources

Original Publication Open Access

Implementing Standardized Patient Caregivers to Practice Difficult Conversations in a Pediatric Dentistry Course

Beau D. Meyer, DDS, MPH*, Bethany Fearnow, Hannah L. Smith, Sarah G. Morgan, Rocio B. Quinonez, DDS, MS, MPH

*Corresponding author: meyer.781@osu.edu

Abstract

Introduction: Standardized patient (SP) methodology has been used in health professional education to help students develop communication, deeper diagnostic reasoning, and critical thinking skills. Few examples demonstrate the use of SPs to practice difficult conversations with pediatric caregivers in the pediatric dentistry literature. The objective of this educational activity was to describe the implementation of three SPs in a pediatric dentistry course for second-year dental students. **Methods:** We developed three SP encounters covering interactions with caregivers of an infant with severe early childhood caries, an adolescent on the path to gender affirmation, and a child with autism and dental caries whose caregiver was resistant to fluoride- and silver-containing dental materials. We describe the case design process, rubric construction and calibration, student debriefing, and pandemic modifications. We evaluated the effectiveness of the implementation by thematic analysis of student reflections following each encounter using a qualitative descriptive framework. **Results:** Eighty-three students completed each encounter. Qualitative analysis showed that students preferred a more realistic encounter by having a child or other distraction present. Students relied on different elements of motivational interviewing depending on the objective of each encounter and the age of the patient. Overall, the SP encounters were well received by students and faculty as an alternative or supplement to traditional student evaluation methods. **Discussion:** We noted a number of lessons learned about implementing SP methodology in pediatric dental education. With these experiences now in place, future evaluations should measure student performance in the SP encounters against student performance during clinical care.

Keywords
Pediatric Dentistry, Standardized Patient, Dentist, Communication Skills, Dental Education

Educational Objectives

By the end of this activity, learners will be able to:

1. Apply motivational interviewing to an infant oral health visit using the Baby Oral Health Program template.
2. Use motivational interviewing to provide oral health counseling during an infant oral health visit.
3. Provide individualized oral health counseling to an adolescent patient from a vulnerable population.
4. Navigate difficult conversations in the context of a preventive oral health visit with parents of an adolescent dental patient.
5. Generate pediatric dental treatment options based on the caregiver interview and clinical information.

Citation:
Meyer BD, Fearnow B, Smith HL, Morgan SG, Quinonez RB. Implementing standardized patient caregivers to practice difficult conversations in a pediatric dentistry course. *MedEdPORTAL*. 2022;18:11201. https://doi.org/10.15766/mep_2374-8265.11201

Introduction

Clinical simulation has been widely used in health professions to teach and assess clinical and physical skills, including communication.[1,2] A recent review found that 73% of simulation studies demonstrated an educational benefit to learners.[3] As an experiential learning activity and methodology, standardized patients (SPs) allow learners to simulate real-life clinical scenarios in a standardized manner. Generally, but not always, SPs are actors trained to portray specific medical conditions to the learner,[4] providing students concrete opportunities to develop the skills required of practicing clinicians such as deeper diagnostic reasoning, physical examination, and communication skills. Considering the limited pediatric dentistry experiences during dental school,[5] SP methodology provides structured, simulated experiences related to pediatric patients and their caregivers in order

Copyright © 2022 Meyer et al. This is an open-access publication distributed under the terms of the Creative Commons Attribution license.

to better prepare students to treat pediatric patients upon graduation.

Within medicine and nursing, SP methodology is an effective educational tool for improving student communication skills and confidence in a variety of scenarios.[6-8] Challenging and uncomfortable conversations with patients are common in health care, and SP methodology can be particularly helpful in training for these scenarios, as they provide realistic practice and direct feedback on communication skills.[8,9] Students describe positive outcomes after experiencing different situations in a controlled setting where direct feedback is available.[9-17] Many studies show the value of SP encounters in health care using standardized empathy or communication assessments employed by a neutral observer.[6,7,9,13,17-21]

Within pediatrics, SP methodology is more readily applied to teach communication strategies when addressing caregivers. Some institutions have included children between the ages of 7 and 16 as SPs to help residents develop better patient-based communication and improve behavior management tactics.[22] After participating in these pediatric SP encounters, learners show improved skills ranging from explaining a treatment plan to caregivers to delivering the news of the death of a child.[23-25] Overall, the use of SP methodology applied to caregivers or children has a positive effect on communication skills and confidence as a part of medical education at both the pre- and postdoctoral levels.[8-10,12,18-22,26,27] Reports calling for increased use of SP methodology for evaluating diagnosis and treatment planning in dental education go back to the 1990s.[28,29] Since then, dental schools have implemented SP encounters in the predoctoral curriculum, mostly with adult patients or interprofessional teams.[30-36] As an introduction to simulation, SP encounters can be utilized in dental education to help students practice patient interviewing and history taking, creating and delivering dental treatment plans, obtaining informed consent, and having difficult conversations about controversial topics such as the use of fluoride, dental materials, or radiography. Much as in other health care fields, current research on SP encounters in dentistry suggests the methodology is a valuable part of dental education, improving provider communication, confidence, and conflict resolution skills.[23,25]

Multiple factors can influence the successful implementation of a simulation program, including support from internal and external stakeholders, access to appropriate resources, and continuous faculty development.[37,38] Fortunately, frameworks exist to guide educators seeking to implement a simulation program in an existing curriculum.[37,39] The most meaningful simulations relate the learning exercise to improved patient outcomes.

At the University of North Carolina at Chapel Hill Adams School of Dentistry (UNC-CH), SP methodology is used as a critical and recurring assessment method, both formative and summative. In 2014, UNC-CH introduced its first two SP encounters into the dental student curriculum. In 2020, students completed 16 SP experiences prior to graduation. With the growing emphasis on experiential learning, the introduction of a newly designed Advocate-Clinician-Thinker curriculum at UNC-CH will provide additional opportunities to pilot and implement new SP experiences as a core component of a student's educational experience.[40] Currently, nearly 40 SP encounters are planned in the new curriculum.

While the use of SP methodology has been well described within nursing and medical education related to pediatric training, its application to pediatric dentistry is relatively unreported. The primary objective of this educational activity was to describe the implementation of low-stakes SP encounters in a pediatric dentistry course for second-year dental students. Specifically, in this publication, we describe the process for implementing SPs from case design to student evaluation, lessons learned for quality improvement, and adaptations forced by the recent pandemic. Additionally, we include a qualitative analysis of student reflections about the new assessments.

Methods

At UNC-CH, the second-year dental students take a course titled Behavior, Communication, and Culture: Children and Developmentally Disabled. The course uses a child developmental framework from infancy through adolescence for examining the interaction between dentists, pediatric patients, and adult caregivers. Teaching emphasizes clinical and personal oral health behavior to set expectations for behavior change as children mature. More broadly, faculty cover topics of social, cultural, and family determinants of oral health. A primary course objective is describing cultural and family determinants of oral health behaviors and care seeking for all children, including those with special health care needs. With support from the Office of Academic Affairs, SP encounters were implemented in the spring 2020 semester as a course evaluation metric.

Designing the SP Experience

The UNC-CH Office of Medical Education has maintained a Clinical Skills and Patient Simulation Center on campus for decades. This center hires SPs and manages them throughout their employment, including background checks, training,

Copyright © 2022 Meyer et al. This is an open-access publication distributed under the terms of the Creative Commons Attribution license.

calibration, and deployment in simulation exercises for health professional students. The clinical space contains 12 exam rooms set up like a traditional medical exam room with two cameras available to record the encounters, one taping the SP and one taping the student. SPs provide consent for recording when hired by the center. Dental students sign a generic electronic media policy that includes the use of video recording for assessment, reflection, and feedback. The recorded videos are stored on university-approved software (CAELearningSpace Enterprise).

The Office of Academic Affairs within UNC-CH provided resources, including money, time, and staff support, for all three encounters. The center charged $20/hour for each SP, with a 2-hour minimum, and each encounter had 12 SPs. Including additional overhead such as equipment use and Clinical Skills and Patient Simulation Center staff, the final cost for three encounters was approximately $4,200. The course director and simulation specialist collaborated for nearly 16 hours per encounter to create detailed case summaries and rubrics.

Prior to the students' first SP encounter, they received an in-person orientation to the simulation clinical environment including an overview of the encounter process (e.g., check-in, pre-encounter prompts, postencounter wrap-up, etc.). Before we used SP methodology in the pediatric dentistry course, students completed three SP encounters with adult patients in an oral medicine course during which they rehearsed history taking and assessing a patient's chief complaint. We oriented the SPs to each case during a 30- to 60-minute session approximately 1 week before the encounters.

Due to time constraints within the curriculum, each encounter lasted 15 minutes per student. We spaced the encounters approximately 3-4 weeks apart and scheduled them during the regularly assigned course time. The course began in January, and students started seeing pediatric patients in clinic that same month, so there was some overlap between the simulated encounters and real-life encounters for certain students.

To begin case development, the course director identified three topic areas pertinent to the course objectives. Each encounter covered course content delivered in preceding weeks, and each encounter was mapped to Commission on Dental Accreditation (CODA) standards for predoctoral dental education.[41] We mapped all encounters to communication standards 2-16 and 2-17, as well as oral hygiene standards 2-22, 2-23, and 2-24.

Implementation

All three encounters contained three pieces of documentation: a detailed patient summary, a brief summary for students, and a communication rubric completed by the SP. The final encounter also included a content rubric evaluation for faculty. For each encounter, we provided the SP with a written summary of the child's history, oral hygiene practices, diet, fluoride exposure, and list of concerns in order to answer anticipated student questions. We provided students with a brief summary of patient demographic information immediately prior to entering the simulation room. Students completed the encounters individually to simulate the provider-caregiver relationship.

Encounter 1 (in person): The first encounter followed the Baby Oral Health Program template, which included using motivational interviewing techniques to cover a variety of pediatric oral health topics (Appendix A).[42] The SP was the caregiver of a 2-year-old boy referred by a pediatrician due to concerns about dark spots on the front teeth. The door note for this encounter contained basic written instructions for the students and provided estimated time lines for each interview topic (Appendix B). We designed each subsequent encounter to increase in difficulty.

Encounter 2 (in person): The second encounter emphasized oral health counseling for an adolescent on the path to gender affirmation (Appendix C). Students were expected to establish trust and foster empathy with the caregiver.[43,44] The SP was the caregiver of the adolescent, who also had an eating disorder. The caregiver was recently divorced over disagreements about the child's gender identity emergence (the mother was more accepting than the father). The door note contained the patient's name, age, and a clinical photo representing dental erosion, for which the students were expected to identify a cause and provide counseling to manage its effects (Appendix D).

Encounter 3 (virtual): As the course progressed, the global coronavirus pandemic caused the school to suspend in-person activities, including SP encounters. Rather than postpone the experience entirely, we adapted the final SP experience to a virtual format.[45] Originally, we had designed the final SP encounter to assess a student's ability to quickly synthesize clinical information and apply preventive, disease management, and behavior management principles to pediatric dental treatment planning for a child with autism whose caregiver was skeptical of using fluoride (Appendix E).[46] As an alternative, we recorded an SP interview between the board-certified pediatric dentist course director and the SP specialist of the simulation center via videoconferencing during the first week of the pandemic shutdowns. The SP was the caregiver of a 6-year-old

Copyright © 2022 Meyer et al. This is an open-access publication distributed under the terms of the Creative Commons Attribution license.

boy with autism who had not been to the dentist in nearly 3 years. The caregiver was also a single parent and was reluctant to use fluoride-containing products or have treatment completed under general anesthesia. Clinical photos were shared with the SP and students (Appendix F). In the recorded interview, any time the SP had a question, the course director responded, “That’s a great question, let me check with my colleagues.” The students played the role of the colleague and were instructed to create a 5- to 10-minute video of themselves responding to the SP caregiver’s questions and concerns, specifically those questions related to prevention, disease management, and behavior management. Additionally, students were asked to provide a prioritized list of treatment options.

We have previously reported results from the video encounter; thus, the results presented here focus on the first two encounters.[45] For those planning to incorporate this educational resource at their institution, the video of the course director interviewing the simulation specialist is included as Appendix G.

Learner Assessment

We considered the SP encounters to be both training and assessment—training in that each encounter provided the learner with an opportunity to practice different interviewing and counseling techniques, assessment in that each encounter provided feedback to the learner from the SPs and from pediatric dentistry faculty.

Three groups of individuals assessed the learners: the SPs, pediatric dentistry faculty (only for the video encounter), and the course director. SPs assessed learner communication skills and professionalism (Appendix H). Pediatric dentistry faculty assessed the quality of the oral health counseling and treatment planning of the adapted video encounter.[45] The course director assessed the quality of counseling in each encounter, as well as providing general debriefing and follow-up after each encounter.

Following each encounter, the SPs evaluated students’ communication skills and professionalism using a checklist rubric adapted from a template provided by the university simulation center (Appendix H). Departmental faculty calibrated the rubrics by reviewing individual criteria and scoring guidance at weekly departmental meetings. Two calibration sessions occurred, one at the time of rubric design (December 2019) and one immediately before grading the final exercise (March 2020). The SPs were trained and calibrated during onboarding, and the rubrics were reviewed during the orientation session for each encounter. The SPs provided written comments and direct feedback in face-to-face debriefings with the students following each encounter.

We instructed students to complete a reflection on their performance and on the exercise itself using reflection prompts (Appendix I). The questions were developed and piloted in other adult SP encounters used at the school, resulting in a brief list of prompts to balance student time and reflection quality. We instructed students to review their recording with a classmate and to use the prompts as discussion starters between them. Then, we asked them to submit written responses to the prompts to the course director in an online form. Due to time and resource constraints, we debriefed during class a week after each encounter in two ways. First, a simulation expert (Bethany Fearnow) shared written feedback with students via email. Second, the course director spent the first 15-20 minutes of class reviewing general feedback from the SPs, sharing faculty observations of the video encounters, and responding to student questions. A facilitators guide is provided in Appendix J.

In our initial implementation of these encounters, we considered them low stakes since they did not constitute a significant portion of the course grade. The student could fail the encounter but still pass the course because course objectives covered broader topics than the SP encounters. We did not want to weight these encounters heavily during this period in the event that they were poorly executed by the course director or poorly received by the students. We considered the assessments for the first two encounters to be formative in that students had opportunities to practice and refine motivational interviewing skills with written and verbal feedback from the SPs, their peers, and the course faculty. The third encounter was considered summative because students were evaluated using the content rubric, which has been described in a prior publication.[45]

Analysis

First, we calculated descriptive statistics for the communication rubrics used by the SPs and summarized them by the five domains outlined in Appendix G: (1) set the stage, (2) obtain the chief complaint and set the agenda, (3) explore the history of present illness, (4) use facilitation skills throughout, and (5) conclude the interview. Second, we performed a qualitative analysis of student performance using written responses to open-ended questions in the reflections as the data-collection instrument. We used the same prompts for each encounter, and we analyzed reflections iteratively between encounters to allow for feedback to be incorporated into the subsequent encounter. Two team members (Danielle Swing and Beau Meyer) with experience using qualitative methodology independently coded

Copyright © 2022 Meyer et al. This is an open-access publication distributed under the terms of the Creative Commons Attribution license.

the free-response questions. The codebook was modified based on inductive themes that emerged during analysis. To enhance trustworthiness, the coders met virtually to review the results and reconcile any discrepancies. We used thematic content analysis and a qualitative descriptive framework for analysis similar to previous work.[47] This evaluation was reviewed by the UNC-CH Office of Human Research Ethics and was determined to be a quality improvement program evaluation exempt from further review (IRB#20-0290).

Results

At UNC-CH, the class of 2022 was composed of 83 students from diverse educational and social backgrounds. Within this class, four students entered dental school with graduate-level degrees ranging from English to public health. The average age of the class was 23 years (range: 19-36 years), and the class had a 1:1 male-to-female ratio. More than 66% of students were White, 10% were African American, 7% were Hispanic, and nearly 75% were in-state residents.

All 83 students completed each encounter and submitted written reflections, and the SPs completed the communication rubric for each student for each encounter. On average, students achieved 95% of the checklist items. Student performance was lowest on the domain of concluding the interview, likely due to time constraints. Student performance on the adolescent encounter (96%) was better than on the infant encounter (93%); nonetheless, students performed higher than 90% on both encounters. Students' two most frequently missed items, regardless of the encounter, were identifying their role as student on the care team (18% did not complete this item) and explaining that they would step out to discuss the case with the supervising dentist (24% did not complete this item). The checklist performance is summarized in the Figure.

In general, students, faculty, and SPs reflected positively on this initial implementation of caregiver simulations. The SPs provided verbal, in-person feedback about the exercises to one of the authors (Bethany Fearnow) immediately following each encounter. The faculty provided feedback to the course director at the weekly faculty meeting following each encounter. One of the main suggestions from students was to increase the realism of each encounter by having the child physically represented in some way, even if it was the sound of a crying or screaming child as a distraction. Due to ethical and legal reasons, it was not possible to have child SPs for the course encounters. However, following these suggestions, clinical photos were added to the second and third encounters.

Student self-reflections are summarized thematically in the Table. In the infant encounter, two important themes that emerged

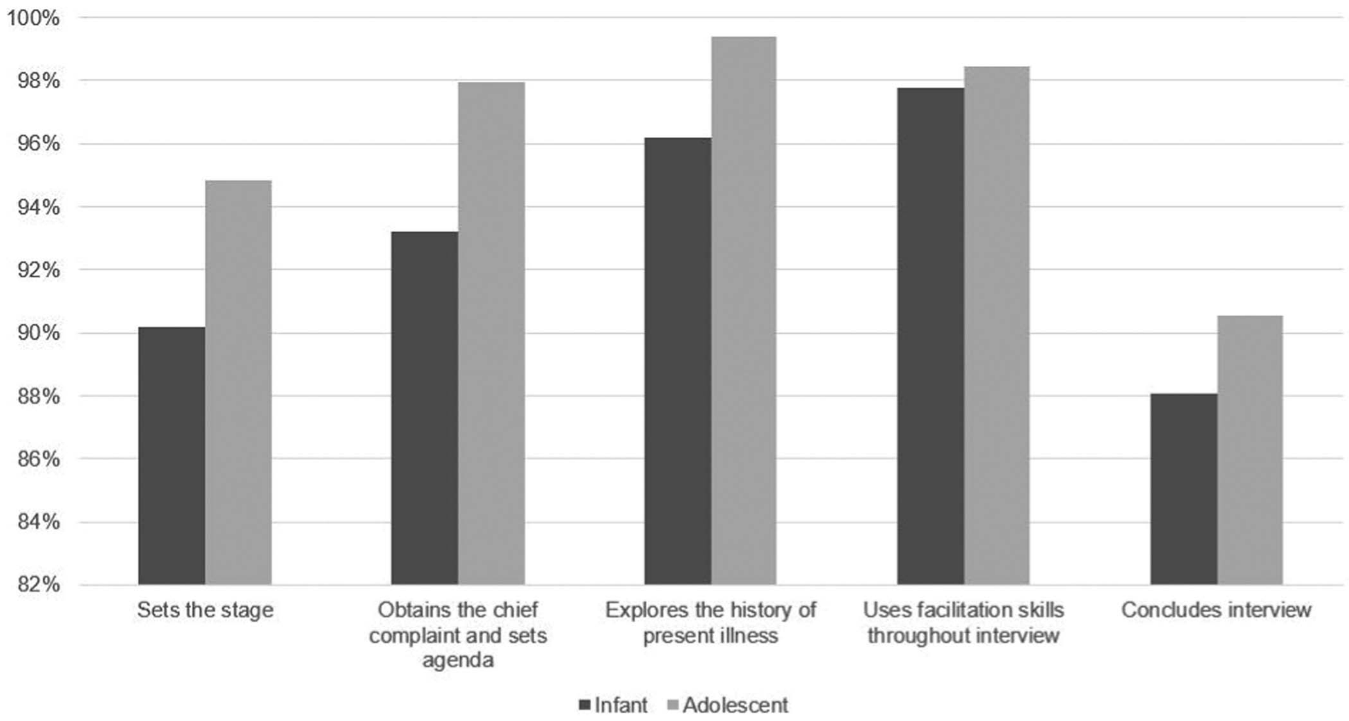

Figure. Summary frequencies (percent completed) of the communication rubric completed by the standardized patients for the 83 students.

Copyright © 2022 Meyer et al. This is an open-access publication distributed under the terms of the Creative Commons Attribution license.

Table. Thematic Analysis of Student Self-Reflections Following Each Encounter

Encounter	Prompt	Theme	Illustrative Quote
1. Infant oral health visit	Name two things that went well.	Building rapport	"I think that I was able to make the patient feel at ease and comfortable. I feel that the environment was very warm and welcoming and [the caregiver] was not afraid to ask questions."
		Completeness of the interview	"The encounter flowed smoothly and covered the main topics that needed to be covered to get an accurate picture of the child's health and caries risk."
	Name two things that were the most challenging.	Balancing competing interests and expectations of the provider and parent	"Striking the right balance between my expectation/hope for change with what the patient/parent appeared to be ready for today." "Making sure that the advice offered to the parent came across as helpful advice from the dentist regarding their child's oral health and not like we were trying to tell them how to parent their child."
		Staying on task	"The most challenging thing for me was staying on task when balancing evoking change talk opportunities with completing the interview."
		Limited experience with children	"I realized during the encounter that I need to practice explaining things to children and their families."
	Name two suggestions for making the infant encounter better.	Improving realism	"Adding a distraction to the infant encounter might be beneficial, as during a real infant encounter we are sure that there are many distractions."
		More time	"There was too much of a time constraint to fully talk with the parent about improving their child's oral hygiene habits."
2. Adolescent new-patient visit	Name two things that went well.	Respectful of sensitive topics	"Maintaining professionalism with a topic I am not particularly comfortable with."
		Making clinical connections	"It was nice identifying a perceived problem from the patient and linking it to a patient behavior. In this case, linking lingual surface erosion to bulimia."
	Name two things that were the most challenging.	Discomfort with sensitive topics	"I also found the overall topic of discussion most difficult. It made me uncomfortable talking about things that can be so sensitive for people, especially when I don't yet know their point of view on the situation."
		Using empathy	"It was challenging to talk with a parent about a really serious situation. Hard to know what to say to the parent and how to empathize when you haven't had the same [life experience]."
		Understanding role in care team	"Talking about bulimia in an adolescent was extremely challenging. I felt sort of helpless suggesting measures we could take in the dental office to help the symptoms of bulimia without really being able to help with the root of the problem."
	Name two suggestions for making the adolescent encounter better.	Increase the challenge	"I think it would have been interesting if the parent had been more 'emotionally invested' so that we were forced to be more empathetic. Even if they had just acted more upset about their daughter's struggle with bulimia, or their divorce, just so that it would force me to work harder to be more compassionate."
		Include an adolescent patient in the room	"Would obviously be more realistic if the child was there (though, this is likely not possible). This situation would force us to better handle potentially uncomfortable situations."

from the students' self-reflections were building rapport with the caregiver (i.e., the SP) even though they may be inexperienced with infant patients and managing time. Students felt that they covered the main topics of the interview, but they noted that time was a major issue. Particularly, some students struggled to stay on task, and their limited experience with pediatric patients made it difficult to both show empathy and provide oral health counseling. Overwhelmingly, the students wanted more time with the SP, as highlighted by the statement, "There was too much of a time constraint to fully talk with the parent about improving their child's oral hygiene habits." In the adolescent encounter, the major theme was discomfort with sensitive topics. Most students were uncomfortable with the sensitive nature of the case topics—both the gender affirmation and the eating disorder. Some students were caught completely by surprise at the notion that an adolescent could be on the path to gender affirmation, and this made it difficult to show empathy and maintain professionalism: "[It's] hard to know what to say to the parent and how to empathize when you haven't had the same [life experience]." During each encounter, students felt they were able to build rapport, be respectful of the patient's situation, and respond to caregiver questions and concerns.

Discussion

In this publication, we describe the implementation of three SP encounters in a pediatric dentistry behavior and communication course for second-year dental students. These encounters replaced the traditional written exam assessments previously used in the course. Students, faculty, and SPs were satisfied with this initial rollout of SP caregiver encounters, including the

Copyright © 2022 Meyer et al. This is an open-access publication distributed under the terms of the Creative Commons Attribution license.

adaptation of the final encounter to distance learning conditions forced by the recent coronavirus pandemic.

It was apparent from students' comments that they were practicing various communication and motivational interviewing skills. In the infant encounter, students leaned more heavily on goal setting and affirmations, whereas in the adolescent encounter, students preferred open-ended questions to elicit information and counsel caregivers. Overall, students were appreciative to have these encounters, and they felt more prepared to enter clinic. With each subsequent encounter, students expressed being and appeared more confident in their responses to SP questions and in the oral health counseling provided.

Lessons Learned

In the role of an SP, a child offers the learner a chance to experience an interaction with a minor patient and their caregiver. The UNC-CH simulation center does not hire children as SPs, which unfortunately prevented an element of realism in each encounter. However, the students offered some interesting suggestions to simulate the child being in the room by having a toy doll, the noise of a child screaming, or a child-sized mannequin. From the students' perspective, the main goal would be to have some distraction to simulate a young child while trying to have an informed conversation with the caregiver.

Another suggestion the students expressed was to include a reference example for how to speak to caregivers of young children. The interview topics were covered in class, and students had an opportunity to practice during in-class sessions. However, this was their first experience in pediatric dentistry, so they really wanted to observe how a faculty member might interview a caregiver before having to complete an interview on their own. Rather fortuitously, the adaptation of the final encounter provided exactly that example when the course director interviewed the simulation specialist. This interview was recorded and will provide a good example for future iterations of the simulation experience.

Student debriefing and feedback provided learners a chance to internalize the learning experience.[34] Even though the reflections, direct SP feedback, and faculty debriefing to the class were helpful for students, 2:1 faculty-and-SP-to-student sessions would provide more personalized feedback. Including both faculty and SPs in the debriefing would provide students with both topical expertise from the faculty and communication expertise from the SP.[48] Unfortunately, time commitments pulled the faculty in different directions, making 83 individual debriefing sessions improbable.

A significant lesson learned during the implementation related to the ethical and legal ramifications of using video recording during SP encounters. The intent of simulation exercises is to place the learner in situations that mimic reality as closely as possible.[49] For the encounters reported in this publication, we obtained specific consent for video recording from the SPs and a generic consent from the students. For future SP encounters at UNC-CH, students will be provided with informed consent for video recording their sessions. This is crucial for ethical and legal reasons, as well as for educational reasons, since the recorded sessions are akin to written exam results in the student's academic record.[49] As when reviewing exam results to correct a misunderstanding, the video recordings allowed students to review specific parts of their encounter that needed improvement.

Limitations and Strengths

The findings from this educational activity should be interpreted in the context of its limitations. Primarily, this was the first iteration of SP encounters in the pediatric dentistry course in the dental student curriculum at a single institution. It is likely that our optimism for this innovation led to confirmation bias in our reflections and overall evaluation. A more rigorous evaluation would have included anonymized evaluators, possibly unaffiliated with the institution but familiar with the methodology and case topics. From a research perspective, the absence of interrater reliability scores limited our ability to accurately quantify learner performance in each encounter. Another limitation was the inability to tie the simulation results to student retention or patient outcomes.[35] Students in this class had not treated pediatric patients, so we could not determine whether the SP methodology used in this course improved student confidence, patient/caregiver satisfaction, or clinical outcomes.

Even though this report is limited in scope, a number of strengths support its findings. Chief among these are the partnership and input from the simulation experts at the UNC-CH simulation center and the institutional support to try new assessment methods. Despite the feeling of success, we identified a number of improvements for future iterations of these simulations. For example, finding ways to include elements of distraction during the infant encounter could improve the sense of realism. In a systematic way, we mapped each encounter to CODA standards. Additionally, the simulations allowed students the opportunity to practice difficult conversations with caregivers,

Copyright © 2022 Meyer et al. This is an open-access publication distributed under the terms of the Creative Commons Attribution license.

a timely topic in dental education.[43,44,46] Lastly, the simulation format was more easily adaptable to the distance learning circumstances forced by the recent pandemic than written exam formats. The flexibility of the SP format provided real-life rehearsals for what students may face in the future health care landscape.

Appendices

A. SP 1 Case.docx
B. SP 1 Door Note.docx
C. SP 2 Case.docx
D. SP 2 Door Note.docx
E. SP 3 Case.docx
F. SP 3 Door Note.docx
G. Example Interview Video.mp4
H. Communication Rubric.docx
I. Reflection Prompts.docx
J. Facilitators Guide.docx

All appendices are peer reviewed as integral parts of the Original Publication.

Beau D. Meyer, DDS, MPH: Assistant Professor, Division of Pediatric Dentistry, The Ohio State University College of Dentistry; ORCID: https://orcid.org/0000-0002-2177-7367

Bethany Fearnow: Curriculum Innovation Consultant, Academic Affairs, University of North Carolina at Chapel Hill Adams School of Dentistry

Hannah L. Smith: Fourth-Year Dental Student, University of North Carolina at Chapel Hill Adams School of Dentistry

Sarah G. Morgan: Fourth-Year Dental Student, University of North Carolina at Chapel Hill Adams School of Dentistry

Rocio B. Quinonez, DDS, MS, MPH: Professor, Division of Pediatric and Public Health, and Associate Dean for Educational Leadership and Innovation, Academic Affairs, University of North Carolina at Chapel Hill Adams School of Dentistry

Acknowledgments
The authors would like to acknowledge Jeff McQueen, a standardized patient training specialist at the University of North Carolina at Chapel Hill (UNC-CH) Office of Medical Education's Clinical Skills, and Patient Simulation Center, for his assistance with case development and facilitating the encounters; Danielle Swing for her assistance with data collection, data organization, and preliminary analysis; and the UNC-CH Academic Support Center for support during case development, curriculum implementation, and standardized patient training.

Disclosures
None to report.

Funding/Support
None to report.

Ethical Approval
The University of North Carolina at Chapel Hill Office of Human Research Ethics deemed further review of this project not necessary.

References

1. Cleland JA, Abe K, Rethans JJ. The use of simulated patients in medical education: AMEE Guide no. 42. *Med Teach*. 2009;31(6): 477-486. https://doi.org/10.1080/01421590903002821
2. Pritchard SA, Denning T, Keating JL, Blackstock FC, Nestel D. "It's not an acting job … don't underestimate what a simulated patient does": a qualitative study exploring the perspectives of simulated patients in health professions education. *Simul Healthc*. 2020; 15(1):21-29. https://doi.org/10.1097/SIH.0000000000000400
3. Williams B, Song JJY. Are simulated patients effective in facilitating development of clinical competence for healthcare students? A scoping review. *Adv Simul (Lond)*. 2016;1:6. https://doi.org/10.1186/s41077-016-0006-1
4. Wind LA, Van Dalen J, Muijtjens AMM, Rethans JJ. Assessing simulated patients in an educational setting: the MaSP (Maastricht Assessment of Simulated Patients). *Med Educ*. 2004;38(1):39-44. https://doi.org/10.1111/j.1365-2923.2004.01686.x
5. Casamassimo PS, Seale NS. Adequacy of patient pools to support predoctoral students' achievement of competence in pediatric dentistry in U.S. dental schools. *J Dent Educ*. 2015; 79(6):644-652. https://doi.org/10.1002/j.0022-0337.2015.79.6.tb05936.x
6. Downar J, McNaughton N, Abdelhalim T, et al. Standardized patient simulation versus didactic teaching alone for improving residents' communication skills when discussing goals of care and resuscitation: a randomized controlled trial. *Palliat Med*. 2017;31(2):130-139. https://doi.org/10.1177/0269216316652278
7. Lorin S, Rho L, Wisnivesky JP, Nierman DM. Improving medical student intensive care unit communication skills: a novel educational initiative using standardized family members. *Crit Care Med*. 2006;34(9):2386-2391. https://doi.org/10.1097/01.CCM.0000230239.04781.BD
8. Downar J, Knickle K, Granton JT, Hawryluck L. Using standardized family members to teach communication skills and ethical principles to critical care trainees. *Crit Care Med*. 2012;40(6):1814-1819. https://doi.org/10.1097/CCM.0b013e31824e0fb7
9. Waltz M, Davis A, Cadigan RJ, Jaswaney R, Smith M, Joyner B. Professionalism and ethics: a standardized patient observed standardized clinical examination to assess ACGME pediatric

Copyright © 2022 Meyer et al. This is an open-access publication distributed under the terms of the Creative Commons Attribution license.

professionalism milestones. *MedEdPORTAL*. 2020;16:10873. https://doi.org/10.15766/mep_2374-8265.10873

10. Findley JC, Schatte D, Power J. A difficult patient encounter: using a standardized patient scenario to teach medical students to treat medication-seeking patients. *MedEdPORTAL*. 2017; 13:10612. https://doi.org/10.15766/mep_2374-8265.10612
11. McClure N, Nelson B, Anderson M, Donnell C, Knox D. Child abuse response simulation for advanced practice nursing students. *Clin Nurse Spec*. 2020;34(4):157-161. https://doi.org/10.1097/NUR.0000000000000529
12. Whitt R, Toussaint G, Binder SB, Borges NJ. Strengthening student communication through pediatric simulated patient encounters. *J Educ Eval Health Prof*. 2014;11:21. https://doi.org/10.3352/jeehp.2014.11.21
13. Marken PA, Zimmerman C, Kennedy C, Schremmer R, Smith KV. Human simulators and standardized patients to teach difficult conversations to interprofessional health care teams. *Am J Pharm Educ*. 2010;74(7):120. https://doi.org/10.5688/aj7407120
14. Ok E, Kutlu FY, Ates E. The effect of standardized patient simulation prior to mental health rotation on nursing students' anxiety and communication skills. *Issues Ment Health Nurs*. 2020;41(3):251-255. https://doi.org/10.1080/01612840.2019.1642427
15. Yeung F, Yuan C, Jackson DS, Chun MBJ. Gone, but not forgotten? Survey of resident attitudes toward a cultural standardized patient examination for a general surgery residency program. *Health Equity*. 2017;1(1):150-155. https://doi.org/10.1089/heq.2017.0016
16. Sack S, Drabant B, Perrin E. Communicating about sexuality: an initiative across the core clerkships. *Acad Med*. 2002;77(11): 1159-1160. https://doi.org/10.1097/00001888-200211000-00025
17. Bowen R, Lally KM, Pingitore FR, Tucker R, McGowan EC, Lechner BE. A simulation based difficult conversations intervention for neonatal intensive care unit nurse practitioners: a randomized controlled trial. *PLoS One*. 2020;15(3):e0229895. https://doi.org/10.1371/journal.pone.0229895
18. Hilgenberg SL, Bogetz AL, Leibold C, Gaba D, Blankenburg RL. De-escalating angry caregivers: a randomized controlled trial of a novel communication curriculum for pediatric residents. *Acad Pediatr*. 2019;19(3):283-290. https://doi.org/10.1016/j.acap.2018.10.005
19. Boykan R, Blair R, Baldelli P, Owens S. Using motivational interviewing to address tobacco cessation: two standardized patient cases for pediatric residents. *MedEdPORTAL*. 2019; 15:10807. https://doi.org/10.15766/mep_2374-8265.10807
20. Tobler K, Grant E, Marczinski C. Evaluation of the impact of a simulation-enhanced breaking bad news workshop in pediatrics. *Simul Healthc*. 2014;9(4):213-219. https://doi.org/10.1097/SIH.0000000000000031
21. Greenberg LW, Ochsenschlager D, O'Donnell R, Mastruserio J, Cohen GJ. Communicating bad news: a pediatric department's evaluation of a simulated intervention. *Pediatrics*. 1999;103(6): 1210-1217. https://doi.org/10.1542/peds.103.6.1210
22. Lane JL, Ziv A, Boulet JR. A pediatric clinical skills assessment using children as standardized patients. *Arch Pediatr Adolesc Med*. 1999;153(6):637-644. https://doi.org/10.1001/archpedi.153.6.637
23. McKenzie CT, Tilashalski K, Abou-Arraj R, Peterson DT, White ML. Students' evaluations of simulations in dentistry: a multiple cohort study at a U.S. dental school. *J Dent Educ*. 2019;83(10): 1142-1150. https://doi.org/10.21815/JDE.019.121
24. Whittle A, Bourne S, Segal S, Serwint JR. Teaching death disclosure: a mixed-method comparison of resident self-assessment and standardized patient assessment. *Acad Pediatr*. 2020;20(5):703-711. https://doi.org/10.1016/j.acap.2020.02.016
25. Beattie BE, Kinney J, Fitzgerald M, et al. Dental and dental hygiene students' perceptions of a standardized patient instructor conflict resolution program. *J Dent Educ*. 2014;78(10): 1397-1404. https://doi.org/10.1002/j.0022-0337.2014.78.10.tb05813.x
26. Kawamura A, Mylopoulos M, Orsino A, Jimenez E, McNaughton N. Promoting the development of adaptive expertise: exploring a simulation model for sharing a diagnosis of autism with parents. *Acad Med*. 2016;91(11):1576-1581. https://doi.org/10.1097/ACM.0000000000001246
27. Rao P, Hill E, Palka C, et al. Improving pediatric resident communication during family-centered rounds using a novel simulation-based curriculum. *MedEdPORTAL*. 2018;14:10733. https://doi.org/10.15766/mep_2374-8265.10733
28. Field MJ, Jeffcoat MK. Dental education at the crossroads: a report by the Institute of Medicine. *J Am Dent Assoc*. 1995; 126(2):191-195. https://doi.org/10.14219/jada.archive.1995.0144
29. Johnson JA, Kopp KC, Williams RG. Standardized patients for the assessment of dental students' clinical skills. *J Dent Educ*. 1990; 54(6):331-333. https://doi.org/10.1002/j.0022-0337.1990.54.6.tb02434.x
30. Rivera J, de Lisser R, Dhruva A, et al. Integrative health: an interprofessional standardized patient case for prelicensure learners. *MedEdPORTAL*. 2018;14:10715. https://doi.org/10.15766/mep_2374-8265.10715
31. Maupome G, Holcomb C, Schrader S. Clinician-patient small talk: comparing fourth-year dental students and practicing dentists in a standardized patient encounter. *J Dent Educ*. 2016;80(11): 1349-1356. https://doi.org/10.1002/j.0022-0337.2016.80.11.tb06220.x
32. Kay CJ, Johnson JA, Kopp KC. Standardized patients for teaching geriatric dentistry. *Spec Care Dentist*. 1994;14(6):229-232. https://doi.org/10.1111/j.1754-4505.1994.tb01071.x
33. Suvinen TI, Messer LB, Franco E. Clinical simulation in teaching preclinical dentistry. *Eur J Dent Educ*. 1998;2(1):25-32. https://doi.org/10.1111/j.1600-0579.1998.tb00032.x

Copyright © 2022 Meyer et al. This is an open-access publication distributed under the terms of the Creative Commons Attribution license.

34. Quick KK. The role of self- and peer assessment in dental students' reflective practice using standardized patient encounters. *J Dent Educ*. 2016;80(8):924-929. https://doi.org/10.1002/j.0022-0337.2016.80.8.tb06172.x

35. Broder HL, Janal M, Mitnick DM, Rodriguez JY, Sischo L. Communication skills in dental students: new data regarding retention and generalization of training effects. *J Dent Educ*. 2015;79(8):940-948. https://doi.org/10.1002/j.0022-0337.2015.79.8.tb05985.x

36. McKenzie CT, Tilashalski KR, Peterson DT, White ML. Effectiveness of standardized patient simulations in teaching clinical communication skills to dental students. *J Dent Educ*. 2017;81(10):1179-1186. https://doi.org/10.21815/JDE.017.075

37. Tekian A, Harden RM, Cook DA, Steinert Y, Hunt D, Norcini J. Managing the tension: from innovation to application in health professions education. *Med Teach*. 2020;42(3):333-339. https://doi.org/10.1080/0142159X.2019.1687871

38. Lemoine JB, Chauvin SW, Broussard L, Oberleitner MG. Statewide interprofessional faculty development in simulation-based education for health professions. *Clin Simul Nurs*. 2015;11(3):153-162. https://doi.org/10.1016/j.ecns.2014.12.002

39. Khamis NN, Satava RM, Alnassar SA, Kern DE. A stepwise model for simulation-based curriculum development for clinical skills, a modification of the six-step approach. *Surg Endosc*. 2016;30(1): 279-287. https://doi.org/10.1007/s00464-015-4206-x

40. Quinonez RB, Wolcott MD, Reside JM, et al. Call for ACTion: transforming dental education at the University of North Carolina at Chapel Hill. *N C Med J*. 2019;80(3):182-185. https://doi.org/10.18043/ncm.80.3.182

41. Commission on Dental Accreditation. *Accreditation Standards for Dental Education Programs.* Commission on Dental Accreditation; 2019. Accessed July 28, 2020.

42. Vamos CA, Quinonez R, Gaston A, Sinton J. Addressing early preventive oral health care among young children: a pilot evaluation of the Baby Oral Health Program (bOHP) among dental professionals. *J Dent Hyg*. 2014;88(4):202-212.

43. Conard LAE, Schwartz SB. Supporting and caring for transgender and gender-expansive individuals in the dental practice. *J Dent Child (Chic).* 2019;86(3):173-179.

44. Macdonald DW, Grossoehme DH, Mazzola A, Pestian T, Schwartz SB. "I just want to be treated like a normal person": oral health care experiences of transgender adolescents and young adults. *J Am Dent Assoc*. 2019;150(9):748-754. https://doi.org/10.1016/j.adaj.2019.03.025

45. Meyer BD, Fearnow B, Quinonez RB. Asynchronous pediatric caregiver simulation in a virtual setting during the COVID pandemic. *J Dent Educ*. 2021;85(suppl 1):1114-1116. https://doi.org/10.1002/jdd.12319

46. Aoun A, Darwiche F, Al Hayek S, Doumit J. The fluoride debate: the pros and cons of fluoridation. *Prev Nutr Food Sci*. 2018;23(3):171-180. https://doi.org/10.3746/pnf.2018.23.3.171

47. Meyer BD, Fearnow B, Wolcott MD. Introducing legislative advocacy into the dental school curriculum: a mixed-methods evaluation. *J Dent Educ*. 2020;84(12):1378-1387. https://doi.org/10.1002/jdd.12336

48. Teker GT, Odabaşı O. Reliability of scores obtained from standardized patient and instructor assessments. *Eur J Dent Educ*. 2019;23(2):88-94. https://doi.org/10.1111/eje.12406

49. Wilson L, Wittmann-Price RA, eds. *Review Manual for the Certified Healthcare Simulation Educator (CHSE) Exam*. Springer; 2015.

Received: June 7, 2021
Accepted: August 31, 2021
Published: January 3, 2022

Copyright © 2022 Meyer et al. This is an open-access publication distributed under the terms of the Creative Commons Attribution license.
